# Risk of Liver and Non-Liver Malignancy in HCV-Infected Patients with Cirrhosis After Direct-Acting Antiviral Treatment

**DOI:** 10.3390/cancers18132079

**Published:** 2026-06-26

**Authors:** Dorota Zarębska-Michaluk, Krystyna Dobrowolska, Robert Flisiak, Kinga Brzdęk, Jakub Janczura, Piotr Rzymski, Michał Brzdęk

**Affiliations:** 1 Department of Infectious Diseases and Allergology, Collegium Medicum, Jan Kochanowski University, 25-516 Kielce, Poland; dorota1010@tlen.pl; 2 Collegium Medicum, Jan Kochanowski University, 25-516 Kielce, Poland; krystyna.dobrowolska98@gmail.com (K.D.); kuba.janczura@gmail.com (J.J.); 3 Department of Infectious Diseases and Hepatology, Medical University of Bialystok, 15-540 Białystok, Poland; robert.flisiak1@gmail.com; 4 Department of Rheumatology and Connective Tissue Diseases, Nicolaus Copernicus Memorial Hospital, 93-513 Lodz, Poland; brzdekinga@gmail.com; 5 Department of Environmental Medicine, Poznan University of Medical Sciences, 60-806 Poznan, Poland; rzymskipiotr@gmail.com; 6 Department of Gastroenterology, Medical University of Lodz, 92-213 Lodz, Poland

**Keywords:** hepatitis C virus, direct-acting antivirals, liver cirrhosis, hepatocellular carcinoma, decompensation, survival

## Abstract

**Background**: Patients with liver cirrhosis remain at a persistent risk of developing hepatocellular carcinoma (HCC) and liver decompensation even after hepatitis C virus (HCV) eradication. **Objectives**: We aimed to assess survival, the occurrence of recompensation and de novo decompensation events, and hepatic and extrahepatic malignancies in patients with liver cirrhosis during long-term observation (LTO) after direct-acting antiviral (DAA) treatment. **Methods**: The study population consisted of 326 consecutive HCV-infected patients with liver cirrhosis treated with DAAs between 1 July 2015 and 30 June 2025 at a single tertiary hepatology center, of whom we followed 298 individuals for a median (IQR) of 4.4 (2.4–8.1) years after treatment completion. **Results**: Men predominated in the study population, with a median age of 60 (48–69) years. The vast majority of patients had at least one comorbid condition. Fifty of them (16.8%) developed malignancies during LTO, including 33 cases of HCC and 17 with extrahepatic malignancies. New cases of HCC were documented as late as 9 years after DAA therapy. Overall, 103 patients (34.6%) died during LTO; 51 deaths occurred in patients with malignancies. Liver recompensation was observed in 27.8% of patients, while 9.9% experienced de novo decompensation events during LTO. The survival curve showed a gradual decline in survival probability from 0.96 at 1–2 years to 0.70 at 5 years and 0.51 at 10 years. **Conclusions**: Patients with cirrhosis should remain under long-term hepatological monitoring after antiviral treatment, as they are still at risk of liver decompensation and the development of de novo HCC.

## Simple Summary

In the era of direct-acting antiviral (DAA) drugs, hepatitis C virus (HCV) can be eliminated in nearly 100% of patients. However, those who have advanced liver disease at the start of antiviral therapy remain at risk of developing liver cancer and liver failure, despite successful treatment. Our study, which included one of the longest up-to-date follow-up cohorts of patients with cirrhosis after DAA treatment, demonstrated the development of new cases of liver cancer as late as 9 years after treatment completion and new episodes of cirrhosis decompensation in 10% of the population. Thus, we document that HCV cure is not the “end of the road” for patients with liver cirrhosis, but that continued structured hepatological follow-up is necessary.

## 1. Introduction

Chronic infection with the hepatitis C virus (HCV), which, according to current World Health Organization estimates, affects approximately 50 million people worldwide, can lead to severe health consequences, causing about 240,000 deaths annually [1]. Among them, cirrhosis of the liver with the risk of decompensation comes in first place; according to available data, it occurs in 15–30% of patients with chronic hepatitis C (CHC) after an infection lasting about 20 years [1]. Patients whose disease has progressed to cirrhosis are at a 2–8% annual risk of developing hepatocellular carcinoma (HCC), which is another substantial and life-threatening hepatic consequence of chronic HCV infection [2]. However, the burden of CHC extends beyond liver-related complications [3]. HCV infection may lead to mixed cryoglobulinemia and associated vasculitis affecting multiple organs [3]. In addition, HCV-infected individuals exhibit increased rates of insulin resistance, diabetes mellitus, and atherosclerosis, contributing to elevated cardiovascular morbidity and mortality [4,5].

Malignancies constitute another important category of extrahepatic conditions associated with chronic HCV infection [6]. Non-Hodgkin lymphomas have the best-documented pathogenetic link to HCV infection through chronic immune stimulation, leading to B-cell proliferation [3]. Other mechanisms by which HCV infection contributes to oncogenesis include chronic inflammation and the virus’s ability to replicate in extrahepatic tissues, causing direct cell damage and promoting malignant transformation [7]. Among solid cancers associated with HCV infection, thyroid, lung, pancreatic, head and neck, and kidney cancer are listed [8,9]. Importantly, the risk of both hepatic and extrahepatic malignancies appears to be particularly elevated in patients with cirrhosis [7,10].

The introduction of direct-acting antivirals (DAAs), including regimens based on sofosbuvir, ledipasvir/sofosbuvir, sofosbuvir/velpatasvir, glecaprevir/pibrentasvir, and ombitasvir/paritaprevir/ritonavir with or without dasabuvir, revolutionized HCV treatment and enabled sustained virological response rates exceeding 95% [10,11,12]. Nevertheless, successful viral eradication does not completely eliminate the risk of adverse clinical outcomes, particularly among patients with established cirrhosis [7,10]. These individuals remain at risk of HCC, liver decompensation, and potentially extrahepatic malignancies. As the population of patients cured of HCV continues to grow, defining their long-term prognosis and follow-up needs has become increasingly important [13,14]. However, real-world studies providing long-term data on survival, liver disease progression, recompensation, and the occurrence of hepatic and extrahepatic malignancies after DAA therapy remain limited. Most published long-term DAA cohorts have focused primarily on individual outcomes, such as HCC incidence or overall survival. In contrast, comprehensive real-world studies simultaneously evaluating long-term hepatic and extrahepatic malignancies, liver recompensation and de novo decompensation, and survival in a well-defined cirrhotic population with follow-up extending close to a decade remain scarce.

Therefore, we conducted a retrospective study of consecutive patients with HCV-related cirrhosis treated with DAAs at a single tertiary hepatology center. The aim was to assess long-term survival, the occurrence of liver recompensation and de novo decompensation, and the incidence of hepatic and extrahepatic malignancies during long-term observation (LTO) following antiviral therapy.

## 2. Materials and Methods

### 2.1. Studied Population and Data Collection

**Figure 1:**
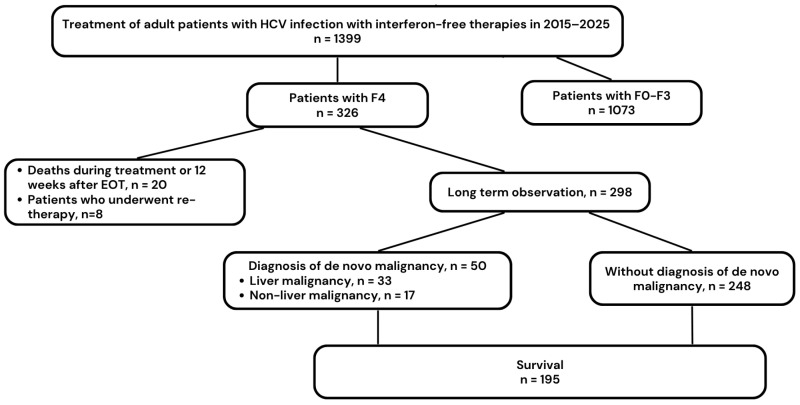
Flowchart of the study. Abbreviations: HCV, hepatitis C virus; EOT, end of treatment; F0–3, fibrosis stages 0 to 3; F4, fibrosis stage 4 (cirrhosis).

The study population consisted of 326 HCV-infected patients with liver cirrhosis among 1399 patients treated with DAAs between 1 July 2015 and 30 June 2025 at a single tertiary hepatology center in the Department of Infectious Diseases in Kielce, Poland. Of these, 298 patients were available for LTO after treatment completion (Figure 1).

Patients were treated under a reimbursed drug program of the National Health Fund in Poland (NHF) in accordance with the international and national recommendations of the Polish Expert Group on HCV and the principles of the drug program [15].

We retrospectively collected patient data from medical records. The dataset included demographic characteristics, comorbidities, HCV genotype (GT), and baseline viral load, co-infection with human immunodeficiency virus (HIV) and hepatitis B virus (HBV), previous anti-HCV treatment, current DAA regimen, laboratory and clinical parameters, including the presence of HCC and a history of orthotopic liver transplantation (OLTx). Steatotic liver disease (SLD) was diagnosed based on ultrasound examination performed before the initiation of DAA therapy. Information on alcohol consumption was also obtained from patients.

### 2.2. Liver Disease Severity Assessment

Liver fibrosis was assessed using shear-wave elastography performed with the Aixplorer system (SuperSonic Imagine, Aix-en-Provence, France). A liver stiffness value ≥ 13 kPa was used to identify patients with probable cirrhosis (F4) [16]. Liver disease severity was additionally evaluated using the Child–Pugh (CP) classification. The classification is based on five clinical and laboratory parameters (serum bilirubin, serum albumin, prothrombin time/INR, ascites, and hepatic encephalopathy), each scored from 1 to 3 points, resulting in a total score ranging from 5 to 15 points. Patients are classified as CP class A (5–6 points), indicating compensated liver disease, or CP class B (7–9 points) and CP class C (10–15 points), indicating decompensated liver disease. SLD was diagnosed based on the presence of hepatic steatosis on abdominal ultrasound, irrespective of its underlying etiology. Alcohol abuse in the past was defined as a self-reported history of alcohol consumption exceeding 140 g/week in women and 210 g/week in men. No patients reported active alcohol consumption at baseline, as abstinence at the time of DAA initiation was a requirement of the national treatment program. Information on the presence of esophageal varices was also collected.

### 2.3. Antiviral Treatment

Patients received genotype-specific or pangenotypic DAA regimens. Treatment effectiveness was assessed by measuring serum HCV RNA 12 weeks after treatment completion. Undetectable HCV RNA at this time point was defined as sustained virological response (SVR).

Safety data were collected throughout the treatment period and for 12 weeks after therapy completion. Information regarding ribavirin (RBV) dose modifications, treatment interruptions, adverse events (AEs), and deaths was recorded. Particular attention was paid to liver-related AEs, including gastrointestinal bleeding, ascites, and hepatic encephalopathy.

### 2.4. Long-Term Observation

After completion of short-term follow-up, defined as the period from DAA initiation until 12 weeks after treatment completion, patients remained under routine clinical observation at the hepatology center as a group at risk for decompensation and HCC. During LTO, patients underwent regular monitoring of liver function and surveillance for HCC according to national and international recommendations, including abdominal ultrasound every six months, with additional imaging when clinically indicated [13,15]. Data collected during LTO included information on decompensation of cirrhosis, recompensation of liver function in patients with baseline liver decompensation, development of de novo HCC, and, if present, the type of treatment and outcome. Only newly diagnosed (de novo) HCC cases were included in the analysis of liver malignancies during long-term observation. HCC occurring in patients with a previous history of HCC was considered recurrent disease and was not classified as de novo HCC. Data on the development of de novo extrahepatic malignancies during LTO, as well as survival and deaths, including dates and causes, were also captured retrospectively. For the purposes of this study, basal cell carcinoma was not classified as an extrahepatic malignancy because of its low metastatic potential and limited impact on survival [17]. No other non-melanoma skin cancers were identified during long-term observation.

### 2.5. Ethical Considerations

We carried out the study with approval from the Bioethics Committee of Jan Kochanowski University in Kielce (Resolution No. 57/2024, dated 25 July 2024). All patients provided informed consent to participate in the therapeutic program in accordance with NHF regulations.

### 2.6. Statistical Analysis

Categorical variables are presented as counts and percentages (*n*, %). Comparisons between categorical variables were performed using the χ^2^ test or Fisher’s exact test, as appropriate. When multiple pairwise comparisons were conducted, *p*-values were adjusted using the Bonferroni correction. Continuous variables are presented as medians with interquartile ranges (IQRs). Because their distributions deviated from normality, as assessed by the Shapiro–Wilk test, comparisons between groups were performed using the Mann–Whitney U test. Multivariable logistic regression analyses were performed to identify independent predictors of failure to achieve sustained hepatic recompensation and de novo liver decompensation during LTO. Variables entered into multivariable models were selected based on clinical relevance and/or significance in univariable analyses. Associations are presented as odds ratios (ORs) with 95% confidence intervals (CIs). Survival probabilities were estimated using the Kaplan–Meier method. Survival curves were generated for the overall study population and according to sex. Differences between groups were assessed using the log-rank test. Hazard ratios (HRs) with 95% CIs were estimated using Cox proportional hazards regression models. A two-sided *p*-value < 0.05 was considered statistically significant. Statistical analyses were performed using Statistica version 13 (StatSoft, Tulsa, OK, USA) and MedCalc version 15.8 (MedCalc Software Ltd., Ostend, Belgium). GraphPad Prism version 5.1 (GraphPad Software, Inc., La Jolla, CA, USA) was used to prepare graphical elements.

## 3. Results

### 3.1. Baseline Characteristics of the Study Population

The baseline cohort consisted of 326 patients with cirrhosis, with a predominance of men (57.4%) and individuals older than 50 years (75.0%; Table S1). Comorbidities were highly prevalent, with SLD being the most common (69.0%). Prior to DAA therapy, HCC was present in 22 patients (6.7%), and non-liver malignancies were documented in 20 patients (6.1%). Within this group, three patients had coexisting HCC and extrahepatic malignancies (breast, prostate, and kidney cancer, respectively).

Regarding viral co-infections, one patient was co-infected with HIV, and three had HBV (all of whom were HBV DNA negative). Evidence of past HBV exposure (anti-HBc positive/HBsAg negative) was found in 18.0% of the population. A history of increased alcohol intake—defined via unverified patient self-declaration as exceeding 140 g weekly for women and 210 g weekly for men—was reported by 25.0% of patients. Baseline laboratory parameters during DAA therapy are detailed in Table S2.

Overall, 71 patients (22%) were categorized as decompensated at the CP scale at the start of therapy, based on clinical symptoms and laboratory values for bilirubin, albumin, and prothrombin time. No patient in the study population had a history of liver transplantation (Table 1).

Among the 20 patients diagnosed with non-liver malignancies prior to DAA initiation, the distribution included lymphoma (*n* = 3), breast cancer (*n* = 3), leukemia (*n* = 2), prostate cancer (*n* = 2), colorectal cancer (*n* = 2), and single cases (*n* = 1 each) of cervical, basal cell, esophageal, kidney, pancreatic, and gastrointestinal stromal cancers, thigh sarcoma, and neuroblastoma.

### 3.2. HCV Infection and Treatment Characteristics

During DAA therapy, 188 patients (57.7%) received a pangenotypic regimen. Among the entire study cohort, sofosbuvir/velpatasvir ± RBV was the most frequently prescribed regimen, used in 124 patients (38%) (Table S3). The effectiveness of DAA therapy in the intent-to-treat analysis was 89.3%. After exclusion of LTFU patients, the SVR rate was 95.4%.

The scheduled treatment was completed by 92.6% of patients (*n* = 302). During therapy and short-term follow-up, 18 patients experienced an increase in ascites, 17 had worsening encephalopathy, and one suffered variceal bleeding. Twenty patients died during treatment or within the 12-week post-treatment follow-up window. Of these deaths, eight occurred in patients with baseline HCC and were related to advanced liver disease progression (seven from liver failure, one from variceal hemorrhage). Consequently, the mortality rate among patients with baseline HCC during this immediate period was 36.4% (8/22) (Table S4). The 20 patients who died during treatment or short-term follow-up were not included in the LTO cohort.

### 3.3. Long-Term Observation Results

After excluding patients who died (20) and those who retreated (8) (we included only the second successful therapy in these cases), 298 patients entered the LTO. We present the detailed characteristics of the LTO population in Table 2.

Six patients in the LTO study did not respond to treatment. Among them, five died before retreatment pangenotypic options became available (within two years after DAA treatment completion), and one woman consequently refused retreatment: she is still alive 4 years after DAA treatment, with no signs of HCC and extrahepatic cancer. Among the six patients who did not achieve SVR, five were male, three were infected with HCV genotype 3, all had esophageal varices, and four had decompensated cirrhosis (Child–Pugh class B or C).

### 3.4. Malignancies and Mortality

In total, 50 out of 298 patients (16.8%) were diagnosed with malignancies during LTO (Table 3).

In this group, 33 were diagnosed with de novo HCC (11.1%), including three patients with HCC/cholangiocarcinoma (CCC); only one patient with newly diagnosed HCC did not respond to DAA therapy. In contrast, all 17 patients who developed extrahepatic malignancies responded to antiviral treatment. We did not observe a recurrence of HCC diagnosed previously during LTO. During LTO, 103 patients (34.6%) died; in 51 cases, death affected individuals with malignant neoplasms, both diagnosed before DAA treatment and who developed de novo during LTO.

Among the 33 patients who developed liver malignancy during LTO, there were 13 women (39.4%) and 9 patients (27.3%) classified as decompensated at the baseline of DAA treatment (CP B or C). When comparing these patients with those who did not develop liver malignancy during LTO—after excluding patients in whom HCC had been diagnosed before treatment—we found no statistically significant differences (Table 4).

**Figure 2:**
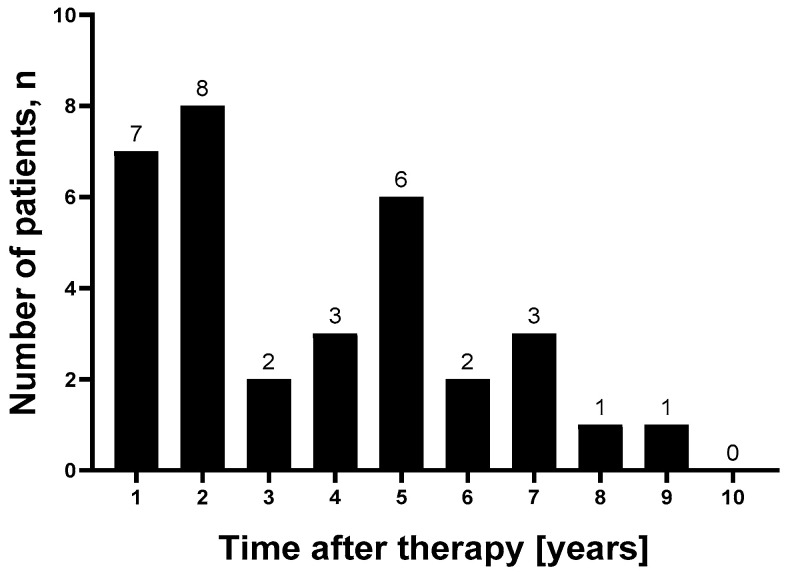
Newly diagnosed HCC in long-term observation.

New cases of HCC diagnosed in LTO over the subsequent years are shown in Figure 2. We documented new diagnoses of liver malignancy as late as 9 years after DAA therapy.

### 3.5. Liver Function Recompensation

Sustained hepatic recompensation—defined according to EASL and Baveno VII criteria as the resolution of hepatic failure symptoms (ascites and/or encephalopathy) lasting at least one year without medical intervention or paracentesis—was achieved by 15 patients who entered LTO with baseline decompensation. All 15 were responders to DAA treatment and permanently transitioned to Child–Pugh Class A during the LTO period. The overall rate of sustained hepatic recompensation was 27.8% (15/54), after excluding two patients from the initial group of 56 Child-Pugh B/C individuals who achieved permanent symptom resolution via OLTx during LTO (both of whom also achieved SVR).

**Figure 3:**
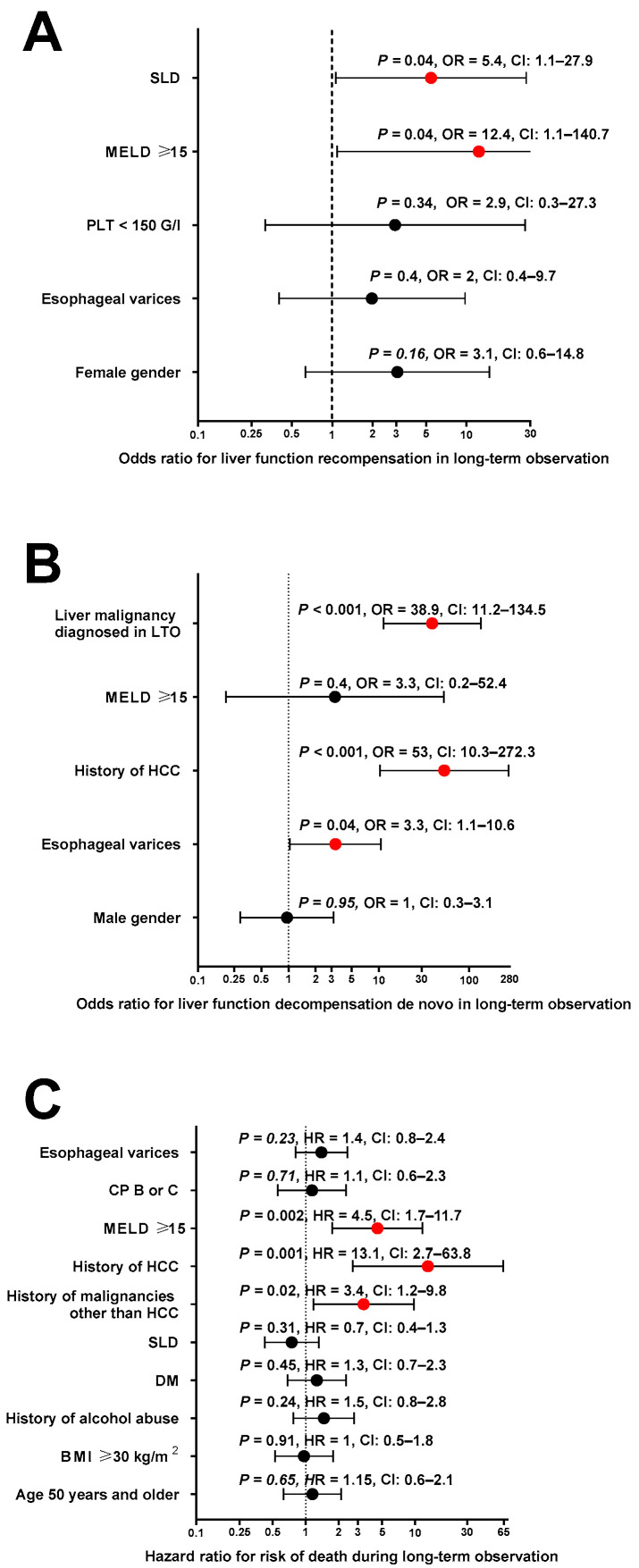
Multivariable analysis of factors associated with (**A**) failure to achieve sustained hepatic recompensation, (**B**) de novo liver function decompensation, and (**C**) risk of death in long-term observation.

Multivariable analysis demonstrated that baseline SLD (OR = 5.4, *p* = 0.042) and a baseline MELD score ≥ 15 (OR = 12.4, *p* = 0.042) were independent risk factors associated with a failure to achieve sustained hepatic recompensation (Figure 3A).

### 3.6. De Novo Decompensation

Twenty-four of 242 patients (9.9%) with compensated cirrhosis at baseline of DAA treatment (A at CP scale) experienced decompensation events in the form of ascites, encephalopathy, or variceal bleeding during LTO; one was a non-responder to DAA therapy. In the multivariable analysis, liver malignancy diagnosed during LTO, history of HCC, and presence of esophageal varices were independently associated with a higher risk of de novo liver function decompensation (OR = 38.9, *p* < 0.001; OR = 53, *p* < 0.001; and OR = 3.3, *p* = 0.044, respectively) (Figure 3B).

### 3.7. Survival in LTO

**Figure 4:**
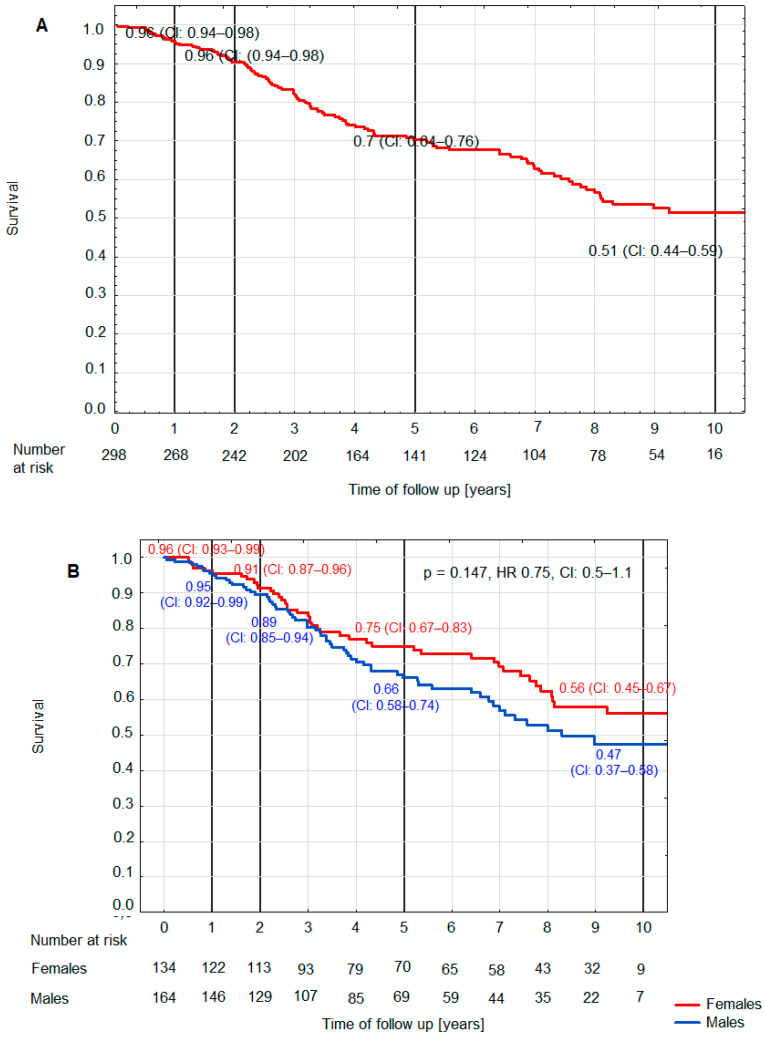
Overall survival in LTO (**A**) and survival in LTO according to gender (**B**). Abbreviations: CI, confidence interval; HR, Hazard ratio.

Kaplan–Meier survival curves revealed a gradual decline in survival probability over time: from 0.96 (95% CI: 0.94–0.98) at 1–2 years of follow-up to 0.70 (95% CI: 0.64–0.76) at 5 years, and 0.51 (95% CI: 0.44–0.59) by year 10, over a median follow-up period of 4.4 years (IQR: 2.4–8.1) (Figure 4A). The difference between females and males did not reach statistical significance (HR 0.75, 95% CI: 0.5–1.1; *p* = 0.15) (Figure 4B).

In the multivariable analysis, MELD ≥ 15 (HR = 4.5; 95% CI: 1.7–11.7; *p* = 0.002), history of HCC (HR = 13.1; 95% CI: 2.7–63.8; *p* = 0.001), and history of malignancies other than HCC (HR = 3.4; 95% CI: 1.2–9.8; *p* = 0.02) were independently associated with an increased risk of death during LTO (Figure 3C).

## 4. Discussion

The introduction of DAA regimens has fundamentally transformed the therapeutic landscape and prognosis for patients with chronic HCV infection. While impressive SVR rates are consistently achieved globally, clinical outcomes remain shaped by baseline patient characteristics [11]. As our single-center retrospective analysis demonstrates, advanced liver cirrhosis remains a primary factor reducing the likelihood of successful viral eradication and compromising overall treatment safety. The present study extends the existing evidence by providing one of the longest real-world follow-up analyses of patients with HCV-related cirrhosis after DAA therapy. Unlike most previous cohorts, our study simultaneously assessed long-term survival, hepatic and extrahepatic malignancies, liver recompensation, and de novo decompensation within a single, consecutively followed population managed according to a uniform institutional protocol.

In our retrospective analysis of a population of patients with cirrhosis, we documented a SVR rate of 95.4%, which, according to data from other real-world evidence (RWE) studies and registration clinical trials, is lower than that observed in patients with less advanced liver disease [11,18,19]. When analyzing the effectiveness of DAA therapy in patients with liver cirrhosis, we should also pay attention to the prevalence of known prognostic factors that are unfavorable for SVR, beyond the cirrhosis itself [20]. These include male gender, which was clearly predominant in our population; GT3 infection, found in 16% of patients; and a history of prior ineffective treatment, which was present in one-quarter of the study group [21,22,23].

An overwhelming majority of our study participants had notable comorbidity burdens, with SLD in 69.0% of cases. Crucially, this prevalence may be underreported; the diagnosis of SLD relied on conventional transabdominal ultrasonography, which typically detects steatosis only when it involves at least 20–30% of the hepatocytes [24]. The data on past alcohol abuse in a quarter of the study population may also have been underestimated, as they were based on patient self-reports rather than objective assessment methods. Both SLD and historical alcohol consumption represent critical, independent metabolic and toxic insults that act synergistically with HCV to accelerate hepatic fibrogenesis and architectural remodeling [24,25].

Extrahepatic malignancies were present at baseline in 6.1% of patients, with breast cancer and B-cell lymphomas emerging as the most frequent diagnoses. The high prevalence of breast cancer can be explained by the fact that it is the most commonly diagnosed cancer in many countries, including Poland [26,27]. There is also a growing body of evidence in the literature pointing to a causal link between breast cancer and HCV infection [28,29]. In B-cell lymphomas, the association with HCV infection is well documented [30,31,32].

The profound risk of HCC in patients with established cirrhosis is a hallmark of the natural history of HCV, driven by ongoing hepatic oncogenesis and cirrhotic tissue remodeling [33]. In our cohort, primary liver cancer was documented in 22 individuals (6.7%) prior to DAA initiation. Crucially, this oncogenic risk persists despite successful viral eradication, which underscores the absolute necessity of lifelong, continuous oncological surveillance in this population [7,13,34]. In strict accordance with international and national recommendations, patients in our study underwent LTO, and we conducted the analysis using retrospectively collected medical record data [15,16].

The primary objective of this study was to estimate the risk of HCC development in patients with cirrhosis in LTO after DAA therapy. According to available data, the annual risk of developing HCC in patients with liver cirrhosis caused by untreated HCV infection ranges from 2% to 8%, depending on the geographic region [2]. In Europe, the 5-year cumulative risk of HCC in HCV-induced cirrhosis over the natural history of the disease was found to be 17% [2,33]. Some reports from the beginning of the DAA era suggested an increased incidence of HCC de novo, as well as recurrence of liver cancer, in patients with cirrhosis following antiviral therapy. However, several subsequent studies demonstrated a reduction in risk following effective therapy [35,36,37,38,39]. This protective effect has also been confirmed in large prospective real-world cohorts. In the multicenter Italian PITER study, patients with HCV-related cirrhosis who achieved SVR following DAA therapy had a significantly lower risk of de novo HCC compared with untreated cirrhotic patients. After adjustment for baseline differences, untreated individuals exhibited a 64% higher risk of HCC, while male sex, older age, ongoing alcohol consumption, HCV genotype 3 infection, thrombocytopenia, and hypoalbuminemia were identified as independent predictors of carcinogenesis [40]. Our study documented an 11% risk of developing HCC during LTO with a median duration of over 4 years. This figure is comparable to the results of an Egyptian study in which 14.5% of cirrhotic patients developed de novo HCC following DAA therapy over a median follow-up period of 5 years [22].

One should note that we did not observe a recurrence of HCC during long-term observation, which supports the findings from other studies [36]. In three patients, oncological evaluation confirmed a rare subtype of liver cancer, namely a combined HCC and CCC [41]. This rare intrahepatic tumor is also epidemiologically and etiologically linked to HCV infection, although the mechanisms of carcinogenesis have not been definitively established [9,42].

One of the key findings of our analysis is that liver cancer can develop in patients with cirrhosis as long as 9 years after successful treatment, which justifies lifelong oncological surveillance in this patient population [13]. We did not identify any risk factors for de novo HCC, which supports continued monitoring of all patients with cirrhosis, regardless of additional characteristics.

Patients with liver cirrhosis, even after successful DAA therapy, are not only at risk of developing HCC, but also liver decompensation [43]. In our study, we observed deterioration of liver function in 10% of patients compensated at the time of antiviral therapy initiation, and only one of them was a non-responder. Liver malignancy in the past, as well as HCC developed post treatment and the presence of esophageal varices, were found to be independent factors of decompensation events, which is in line with conclusions from other studies [44]. In a study involving 193 Egyptian patients with HCV-induced liver cirrhosis—the majority of whom were men (nearly 70%), like in our population—a 17.6% rate of de novo decompensation was documented during a 5-year follow-up period following successful DAA therapy [22].

At the other extreme were patients who had decompensated cirrhosis at the beginning of DAA treatment and experienced recompensation during LTO. In our study, they constituted 28%. This value is lower than that reported in a recently published analysis covering 10 European centers with over 2500 patients, in which compensation was recorded in 36.6% [45]. On the other hand, a recompensation rate of 24.7%, comparable to our results, was documented in a study involving over 1000 patients followed for 4 years after therapy [21]. In our study population, the presence of SLD and a MELD score ≥ 15 significantly reduced the chances of recompensation.

Evaluating overall survival over an extended temporal window was a cornerstone of this analysis. The probability of survival declined incrementally from 0.96 at years 1–2, to 0.70 at 5 years, and ultimately to 0.51 at 10 years post-treatment. Although females demonstrated a favorable survival trend compared with males, this difference did not reach statistical significance. In contrast, a Brazilian study reported lower survival rates of 93% and 66% at 1 and 3 years, respectively [46,47]. However, this study included only patients with decompensated cirrhosis, which may explain why the values were lower than those obtained in our study. On the other hand, a study from the Netherlands, with a median follow-up period after treatment of 8.4 years, showed a significantly higher cumulative survival rate 10 years after antiviral therapy: 91% for successful treatment and 74% for unsuccessful treatment with interferon and ribavirin [47]. One should note, however, that only 54% of the analyzed population had liver cirrhosis [47], while the remaining patients had advanced fibrosis. Consistent with contemporary RWE, our multivariable Cox regression confirmed that a history of HCC, extrahepatic malignancies, and a baseline MELD score ≥ 15 are potent, independent negative predictors of long-term survival [22,44,46,48].

We acknowledge several limitations inherent to our study, chiefly its retrospective design and reliance on historical medical records, which may introduce the possibility of unrecorded clinical data. Quantification of alcohol intake relied on subjective patient recall, and behavioral data regarding cigarette smoking, a known cofactor in extrahepatic oncogenesis, were unavailable. Additionally, dynamic longitudinal shifts in MELD scores, laboratory parameters, and non-invasive liver stiffness measurements during LTO were not modeled. A comprehensive analysis of HCC-related biomarkers was not feasible because AFP measurements were not available for all patients, and PIVKA-II was not routinely assessed. Therefore, potential associations between biomarker profiles and subsequent HCC development could not be evaluated. As this was a retrospective real-world study without linkage to a national cancer registry, under-ascertainment of extrahepatic malignancies cannot be completely excluded, although follow-up data were retrieved from a comprehensive electronic medical record system integrating both inpatient and outpatient care. Another limitation is the lack of a contemporaneous control group. Because the study consisted almost exclusively of patients who achieved SVR following DAA therapy, we were unable to compare HCC incidence with untreated patients, patients who did not achieve SVR, or the general population. Therefore, comparisons with the natural history of HCV-related cirrhosis rely on previously published studies. Although active alcohol consumption was an exclusion criterion for DAA therapy and patients also declared abstinence during long-term follow-up, alcohol exposure was based exclusively on self-report, as no objective method of verification was available. Furthermore, detailed information on the duration of previous alcohol abuse and the length of abstinence before treatment initiation was unavailable. Therefore, the potential influence of alcohol consumption and cessation on liver recompensation and other long-term outcomes could not be fully assessed. Although baseline metabolic characteristics, including BMI, diabetes, steatotic liver disease, and hyperlipidemia, were collected and analyzed, longitudinal changes in metabolic status and liver function parameters (including MELD score, laboratory tests, and non-invasive liver stiffness measurements) were not systematically assessed. Consequently, the impact of temporal changes in these variables on long-term clinical outcomes could not be evaluated. Another limitation is the lack of systematic longitudinal data on the evolution of extrahepatic HCV-related manifestations after DAA therapy, which precluded assessment of their long-term dynamics following viral eradication. Furthermore, information on smoking status and alcohol consumption during long-term follow-up was not systematically available. Likewise, detailed longitudinal data on metabolic risk factors and changes in liver function over time were lacking. These unmeasured variables may have influenced the risk of hepatic and extrahepatic malignancies, liver disease progression, and survival, and therefore should be considered when interpreting the results of this retrospective study. Because this was a single-center study conducted in Poland, caution is warranted when extrapolating our findings to other populations. Differences in ethnicity, HCV genotype distribution, prevalence of metabolic dysfunction and alcohol-related liver disease, access to antiviral therapy, and long-term HCC surveillance strategies may influence the incidence of hepatic complications and survival after DAA therapy [2]. Therefore, multicenter studies including geographically and ethnically diverse populations are needed to validate the generalizability of our findings.

Despite these limitations, the primary strength of our study lies in its highly comprehensive, consecutive follow-up of liver function, survival, and dual oncological profiles (hepatic and extrahepatic) for all eligible patients treated at a single, tertiary academic hepatology center since the dawn of the interferon-free era. Because all patients were treated and monitored under an identical institutional protocol, inter-center management bias was eliminated. To our knowledge, this study represents one of the first comprehensive, long-term real-world analyses from Central and Eastern Europe to simultaneously evaluate survival, de novo HCC, extrahepatic malignancies, and hepatic recompensation dynamics in a dedicated cirrhotic population following DAA therapy.

## 5. Conclusions

Successful DAA therapy dramatically stabilizes liver disease, but does not eliminate the long-term clinical risks associated with pre-existing hepatic cirrhosis. Patients with chronic HCV who have transitioned to liver cirrhosis prior to antiviral treatment require indefinite, structured, long-term hepatological follow-up. Even after achieving SVR, these individuals carry a persistent risk of de novo hepatic decompensation and the development of primary liver malignancies. Because de novo hepatocellular carcinoma can manifest nearly a decade (up to 9 years) following successful viral eradication, and because baseline metabolic comorbidities such as SLD and higher MELD scores (≥15) significantly impair the liver’s capacity for functional recompensation, oncological and clinical surveillance must remain universal and lifelong, irrespective of successful viral cure.

## Figures and Tables

**Table 1:** Characteristics of liver disease in cirrhotic patients.

Parameter	Patients, *n* = 326
Documented esophageal varices, *n* (%)	133 (40.8)
Child-Pugh at the start of therapy, *n* (%)	
B	57 (17.5)
C	14 (4.3)
MELD ≥ 15, *n* (%)	32 (9.9)
HCC diagnosis, *n* (%)	22 (6.7)
OLTx in the past, *n* (%)	0

Abbreviations: MELD, Model for End-Stage Liver Disease; HCC, hepatocellular carcinoma; OLTx, orthotopic liver transplantation.

**Table 2:** Baseline characteristics of population LTO.

Parameter	LTO Population, *n* = 298
Gender, females/males, *n* (%)	134 (45)/164 (55)
Age [years], median (IQR)	60 (48–69)
Age ≥ 50 years old, *n* (%)	218 (73.1)
BMI, kg/m^2^, median (IQR)	26.4 (23.6–30.3)
Comorbidities, *n* (%)	
Any comorbidity	293 (98.3)
Hypertension	153 (51.3)
Diabetes	69 (23.2)
Renal disease	28 (9.4)
Autoimmune disease	20 (6.7)
Steatotic liver disease	204 (68.5)
Non-liver malignancies before DAA therapy	17 (5.7)
Concomitant medications, *n* (%)	259 (86.9)
HCC diagnosis before DAA therapy, *n* (%)	14 (4.7)
HIV co-infection, *n* (%)	0
HBV co-infection (HBsAg positive), *n* (%)	3 (1)
Anti-HBc total positive, HBsAg negative, *n* (%)	52 (17.4)
Alcohol abuse in the past, *n* (%)	69 (23.2)
Child-Pugh at the start of DAA therapy, *n* (%)	
Class B	46 (15.4)
Class C	10 (3.4)
MELD score ≥ 15 at start of DAA therapy, *n* (%)	27 (9.1)
Non-SVR, *n* (%)	6 (2)

Abbreviations: BMI, body mass index; DAA, direct-acting antivirals; HBV, hepatitis B virus; HIV, human immunodeficiency virus; HCC, hepatocellular carcinoma; IQR, interquartile range; MELD, Model for End-Stage Liver Disease; SVR, sustained virologic response; HBc, hepatitis B core antibody; HBsAg, hepatitis B surface antigen.

**Table 3:** Malignancies and deaths during long-term observation in patients with cirrhosis.

Parameter	LTO Population, *n* = 298
New diagnosis of malignancy during LTO, *n* (%)	50 (16.8)
HCC diagnosed in LTO, *n* (%), (including HCC/CCC)	33 (11.1)
Extrahepatic malignancies diagnosed in LTO, *n* (%)	17 (5.7)
Lymphoma	4 (1.3)
Uterine cancer	2 (0.7)
Pancreatic cancer	2 (0.7)
Breast cancer	2 (0.7)
Basal cell cancer	2 (0.7)
Glioma	1 (0.3)
Prostate cancer	1 (0.3)
Colorectal cancer	1 (0.3)
Thyroid cancer	1 (0.3)
Disseminated cancer of unknown origin	1 (0.3)
OLTx during LTO, *n* (%)	8 (2.7)
Deaths during LTO, *n* (%) *	103 (34.6)
Due to the HCC present at baseline of DAA	10 (3.4)
Death in LTO due to HCC/CCC diagnosed in LTO	21 (7)
Due to a non-liver malignancy present at baseline of DAA	9 (3)
Due to a non-liver malignancy diagnosed in LTO	11 (3.7)
Due to liver decompensation without HCC	14 (4.7)

Abbreviations: CCC, cholangiocarcinoma; DAA, direct-acting antivirals; HCC, hepatocellular carcinoma; LTO, long-term observation; OLTx, orthotopic liver transplantation. * Deaths during LTO are among the 298 patients who entered LTO; 20 additional deaths occurred before LTO (see text).

**Table 4:** Comparison of patients with and without a diagnosis of liver malignancy during long-term observation after exclusion of patients with baseline HCC.

Parameter	Liver Malignancy, *n* = 33	Without Liver Malignancy, *n* = 251	*p*
Time of LTO [years], median (IQR)	5.6 (3.1–7.3)	4.5 (2.4–8.4)	0.84
Gender, females/males, *n* (%)	13 (39.4)/20 (60.6)	118 (47)/133 (53)	0.41
Age [years], median (IQR)	61 (54–66)	60 (46–69)	0.72
Age ≥ 50 years old, *n* (%)	28 (84.8)	176 (70.1)	0.08
BMI, kg/m^2^, median (IQR)	27 (24.2–30.1)	26.4 (23.4–30.1)	0.78
Comorbidities, *n* (%)			
Any comorbidity	32 (97)	247 (98.4)	0.55
Hypertension	16 (48.5)	128 (51)	0.79
Diabetes	7 (21.2)	61 (24.3)	0.7
Renal disease	3 (9.1)	24 (9.6)	>0.99
Autoimmune disease	3 (9.1)	17 (6.8)	0.71
Steatotic liver disease	24 (72.7)	169 (67.3)	0.53
Hyperlipidemia	11 (33.3)	63 (25.1)	0.26
Non-liver malignancies	2 (6.1)	14 (5.6)	>0.99
Concomitant medications, *n* (%)	32 (97)	213 (84.9)	0.06
HIV co-infection, *n* (%)	0	0	NA
HBV coinfection (HBsAg(+), *n* (%)	1 (3)	2 (0.8)	0.35
Anti-HBc total(+), HBsAg(−), *n* (%)	6 (18.2)	44 (17.5)	0.93
Alcohol abuse in the past, *n* (%)	8 (24.2)	57 (22.7)	0.84
GT, *n* (%)			0.88
1a	0	1 (0.4)	
1b	28 (84.8)	187 (74.5)	
1	1 (3)	21 (8.4)	
3	4 (12.1)	37 (14.7)	
4	0	2 (0.8)	
6	0	1 (0.4)	
Non determined	0	2 (0.8)	
Acites at baseline of DAA therapy, *n* (%)	6 (18.2)	26 (10.4)	0.37
Encephalopathy at baseline of DAA therapy, *n* (%)	3 (9.1)	8 (3.2)	0.17
CP at baseline of DAA therapy, *n* (%)			
Class B	8 (24.2)	36 (14.3)	0.14
Class C	1 (3)	8 (3.1)	>0.99
MELD score ≥ 15, *n* (%)	6 (18.2)	20 (8)	0.47
SVR (PP)	31/32 (96.9)	246/249 (98.8)	0.39

Abbreviations: BMI, body mass index; CP, Child–Pugh score; DAA, direct-acting antivirals; GT, genotype; HBV, hepatitis B virus; HIV, human immunodeficiency virus; IQR, interquartile range; MELD, Model for End-Stage Liver Disease; SVR, sustained virologic response; PP, per-protocol.

## Data Availability

The datasets used and analyzed during the current study are available from the corresponding author on reasonable request.

## Supplementary Materials

The following supporting information can be downloaded at: https://www.mdpi.com/article/10.3390/cancers18132079/s1, Table S1: Baseline characteristics of the study population; Table S2: Baseline laboratory parameters in patients with F4; Table S3: Characteristics of liver disease, HCV infection, and antiviral treatment; Table S4: Treatment safety.

## Abbreviations

The following abbreviations are used in this manuscript:

AE	Adverse Event
anti-HBc	Antibody to Hepatitis B Core Antigen
CCC	Cholangiocarcinoma
CHC	Chronic Hepatitis C
CI	Confidence Interval
CP	Child-Pugh
DAA	Direct-Acting Antiviral
EASL	European Association for the Study of the Liver
GT	Genotype
HBV	Hepatitis B Virus
HBV DNA	Hepatitis B Virus Deoxyribonucleic Acid
HCC	Hepatocellular Carcinoma
HCV	Hepatitis C Virus
HCV RNA	Hepatitis C Virus Ribonucleic Acid
HIV	Human Immunodeficiency Virus
HR	Hazard Ratio
IFN	Interferon
IQR	Interquartile Range
LTFU	Lost to Follow-Up
LTO	Long-Term Observation
MELD	Model for End-Stage Liver Disease
NHF	National Health Fund
OLTx	Orthotopic Liver Transplantation
OR	Odds Ratio
RBV	Ribavirin
RNA	Ribonucleic Acid
RWE	Real-World Evidence
SLD	Steatotic Liver Disease
SVR	Sustained Virological Response
WHO	World Health Organization
