# Supplementary material for: Risk of Liver and Non-Liver Malignancy in HCV-Infected Patients with Cirrhosis After Direct-Acting Antiviral Treatment
Source: Cancers (Basel). 2026 Jun 26;18(13):2079. doi: 10.3390/cancers18132079 (PMC13359568; doi:10.3390/cancers18132079)
Supplement: Supplementary file 1 [file cancers-18-02079-s001.zip › cancers-4387481-supplementary.pdf]

Table S1. Baseline characteristics of the study population.

Parameter	Patients F4, n=326
Gender, females/males, n (%)	140 (43)/186 (57)
Age [years], median (Q1-Q3)	60 (49-68)
Age ≥50 years old, n (%)	242 (74.2)
BMI, kg/m^3, median (Q1-Q3)	26.36 (23.54-30.12)
Comorbidities, n (%)	
Any comorbidity	321 (98.5)
Hypertension	161 (49.4)
Diabetes	81 (24.8)
Renal disease	35 (10.7)
Autoimmune disease	56 (17.2)
Steatotic liver disease	225 (69)
Obesity (BMI ≥30 kg/m^3)	85 (26)
Non-liver malignancies	20 (6.1)
Concomitant medications, n (%)	286 (87.7)
HCC diagnosis, n (%)	22 (6.7)
HIV coinfection, n (%)	1 (0.3)
HBV coinfection (HBsAg positive), n (%)	3 (0.9)
Anti-HBc total positive, HBsAg negative, n (%)	59 (18.1)
Alcohol abuse in the past, n (%)	80 (24.5)

Abbreviations: BMI, body mass index; HBV, hepatitis B virus; HIV, human immunodeficiency virus; HCC, hepatocellular carcinoma; Q1–Q3, first to third quartile (interquartile range);

Table S2. Baseline laboratory parameters in patients with F4.

Parameter	**Patients F4, n=326**
ALT (IU/L), median (Q1-Q3)	78 (51–119)
Albumin (g/dL), median (Q1-Q3)	3.6 (3.3–3.95)
Bilirubin (mg/dL), median (Q1-Q3)	1.1 (0.8–1.6)
Haemoglobin (g/dL), median (Q1-Q3)	13.7 (12.2–14.7)
Platelets (*1000/μL), median (Q1-Q3)	98 (71–144)
Creatinine (mg/dL), median (Q1-Q3)	0.84 (0.7–1)
INR, median (Q1-Q3)	1.2 (1.1–1.3)
HCV RNA, x10^6 IU/mL, median (Q1-Q3)	0.6 (0.2-1.6)

Abbreviations: ALT, Alanine Aminotransferase; HCV, Hepatitis C Virus; INR, International Normalized Ratio; Q1–Q3, first to third quartile (interquartile range); IU, International Unit; RNA, Ribonucleic Acid;

SI conversion factors:
To convert ALT to μkat/l, multiply by 0.0167.
To convert albumin to g/l, multiply by 10.
To convert bilirubin to μmol/l, multiply by 17.1.
To convert hemoglobin to g/L, multiply by 10.
To convert creatinine to μmol/l, multiply by 88.4.
To convert HCV RNA to copies/mL, multiply by 5.6.

Table S3. Characteristics of liver disease, HCV infection, and antiviral treatment.

Parameter	Patients F4, n=326
HCV RNA, x10^6 IU/mL, median (Q1-Q3)	0.6 (0.2-1.6)
Genotype, n (%)	
1	25 (7.7)
1b	242 (74.2)
1a	1 (0.3)
2	0
3	52 (16)
4	3 (0.9)
5	0
6	1 (0.3)
Not determined	2 (0.6)
History of antiviral therapy, n (%)	
Treatment-naïve	244 (74.8)
Current treatment regimen, n (%)	
Genotype-specific treatment regimens	138 (42.3)
SOF/LDV±RBV	56 (17.2)
OBV/PTV/r±DSV±RBV	51 (15.6)
GZR/EBR±RBV	23 (7.1)
SOF+SMV±RBV	3 (0.9)
ASV+DCV	5 (1.5)
Pangenotypic regimens	188 (57.7)
SOF+RBV	18 (5.5)
SOF+DCV±RBV	1 (0.3)
SOF/VEL±RBV	124 (38)
GLE/PIB	42 (12.9)

Abbreviations: HCV, hepatitis C virus; RNA, ribonucleic acid; Q1–Q3, first to third quartile (interquartile range); SOF, sofosbuvir; LDV, ledipasvir; RBV, ribavirin; OBV, ombitasvir; PTV/r, paritaprevir/ritonavir; DSV, dasabuvir; GZR, grazoprevir; EBR, elbasvir; SMV, simeprevir; ASV, asunaprevir; DCV, daclatasvir; VEL, velpatasvir; GLE, glecaprevir; PIB, pibrentasvir.

SI conversion factors:
To convert HCV RNA to copies/mL, multiply by 5.6.

Table S4. Treatment safety.

Parameter	Patients F4, n=326
Treatment course, n (%)	
According to the schedule	302 (93)
Therapy modification (RBV dose)	9 (2.8)
Therapy discontinuation	15 (4.6)
Patients with at least one AE, n (%)	87 (27)
Weakness/fatigue	32 (10)
Anemia	28 (8.6)
AEs of particular interest in cirrhotic patients, n (%)	
Ascites	18 (5.5)
Hepatic encephalopathy	17 (5.2)
Gastrointestinal bleeding	1 (0.3)
Serious adverse events, n (%)	35 (10.7)
Death, n (%)	20 (6.1)

Abbreviations: AE, adverse event; RBV, ribavirin
